# Patterns of Third-Molar-Pericoronitis-Related Pain: A Morphometrical Observational Retrospective Study

**DOI:** 10.3390/healthcare11131890

**Published:** 2023-06-30

**Authors:** Dafne Chisci, Stefano Parrini, Nicola Baldini, Glauco Chisci

**Affiliations:** Oral Surgery School, Dentistry and Dental Prosthodontics, Department of Medical Biotechnologies, University of Siena, 53100 Siena, Italy; dafne.chisci@studio.unibo.it (D.C.);

**Keywords:** extraction, headache, pain, pathology, pericoronitis, surgery, third molar

## Abstract

Background: Mandibular third molar (M3M) removal and the management of postoperative complications represent a common matter of interest in oral and maxillofacial surgery. Pain represents a great symptom for patients affected by pericoronitis and it is the most common indication for third molar removal. The aim of the present article is to search for patterns of pre-operative pain in patients before undergoing third molar surgery and to test for a relation between some patterns of symptoms, such as pain intensity, site of symptomatic tooth, and referred area of pain. Methods: This retrospective observational study enrolled a total of 86 patients, aged (mean ± SD) 34.54 ± 13.62 years (range 17–78 years), scheduled for outpatient third molar extraction at the Oral Surgery School, Department of Medical Biotechnologies, Policlinico “Le Scotte”, University of Siena. Pericoronitis and pain were the symptoms of the patients and the indication of extraction. Inclusion criteria were the presence of partially impacted third molars, confirmed with a preoperative panoramic radiograph, and preoperative pain. Exclusion criteria were known neurological disease (such as previous trigeminal or facial nerve injuries), impaired communicative or cognitive disease, diagnosed diabetes mellitus, and oral surgical intervention within 30 days before data collection. Patients were visited and asked to answer a morphometric analytic questionnaire about their perception of pain referred to the third molar. Analyses were performed on statistical evaluation on age, age ranges, patient gender, prior third molar extraction, site of pericoronitis, pain score (1–10), and pain area. Two-tailed *p* values of less than 0.05 were considered significant if not otherwise specified. Results: No correlations were found between age, gender, previous extraction, tooth site (maxillar on mandible), pain score, and pain area. Patterns of third molar pericoronitis pain among 86 patients were reported. A significant correlation was found between pain score and pain area (*p* = 0.0111, *r*_s_ = 0.3131). Conclusions: Pain intensity has indeed some kind of responsibility in determining the orofacial distribution of pain. The pain area referral patterns of the present article could be considered as a pain model resulting from the pericoronitis of maxillar and mandibular third molars.

## 1. Introduction

Third molar removal is one of the most common interventions in oral and maxillofacial surgery. The most frequent pathology of the third molar is dysodontiasis and pericoronitis; dysodontiasis regards alterations related to the tooth inclusion, the lack of dragging competence of the periodontal ligament, and probably an eruptive deficit during dental root development [1]. This pathology may affect both the maxillary and mandibular third molar.

Pericoronitis is a typical inflammatory pathology of the impacted or partially impacted third molar that influences the quality of life of the patients before the extraction of the tooth, more commonly in the 20s and 30s [2]. 

The common symptoms of third molar pathology are pain, swelling, and trismus; a characteristic of this pathology is the difficulty for the patient to refer to the upper or lower tooth, as trigeminal innervation often confuses the patient due to the maxillary and mandibular branches [3]. The impaction of third molars is a common condition for most of the patients, and among the symptoms, pain is the most common indication for third molar (3M) surgery [4]. The preoperative symptoms of third molar pathology are commonly similar to postoperative symptoms during recovery after third molar surgery, especially with regard to the lower third molars; upper third molars appear to be lesser in symptom after tooth extraction [3].

As important as the pre-operative symptoms of the third molar, the post-operative conditions after third molar surgery raise relevant issues; several studies have underlined the correlation of many factors (i.e., preoperative pain, decay, periodontitis, age, oxidative stress, gender, and anxiety) with oral disability and severe pain after third molar surgery [5,6,7,8]. Furthermore, many authors have studied the impact of third molar surgery in post-operative pain and health-related quality of life [2,9]; the contact of the mandibular third molar and inferior alveolar nerve; and the time and difficulty of extraction directly related to postoperative pain and reduced health-related quality of life [10].

Apart from correlations with postoperative complications, the entity and facial distribution of preoperative pain due to third molar pericoronitis, in our opinion, has often been underestimated in the international literature. We hypothesize that this condition may be due to the fact that third-molar-referred preoperative pain has not been studied in detail, and a better definition could lead to a more confident diagnosis and a better understanding of the pathology.

With regard to pericoronitis pathology, Caymaz and Buhara in their paper reported evidence of a positive association between the amount of dental plaque and third molar pericoronitis; therefore, they encouraged the improvement of oral hygiene and control of dental plaque in order to prevent third molar pericoronitis [11]. The international literature underlines some aspects that may facilitate the appearance of pericoronitis: Ye et al. reported that soft tissue impaction and vertically angulated teeth were more associated with pericoronitis than other impactions [12]; Galvão et al. in their interesting systematic review and meta-analysis confirmed that vertically angulated teeth presented pericoronitis more frequently, but also position A from the Pell & Gregory classification [13,14]. Singh et al. reported pericoronitis more commonly in females and in distoangular partially impacted mandibular third molars class II and position B from the Pell & Gregory classification [14,15]. Some years later, Singh et al. conducted an interesting cone beam study to evaluate third molar position and pericoronitis, and reported that mesioangular impactions were most commonly observed with pericoronitis [16]. Bradshaw et al. in their well-documented research reported the improvement of health-related quality of life in patients with minor symptoms of pericoronitis, too: the removal of the third molar affected by major or minor pericoronitis appears to be a reliable strategy to improve health-related quality of life [17].

Pericoronitis appears to be a bacterial infection that commonly evokes the most common pain in third molar impaction; however, a recent article reported the presence of viral infection in pericoronitis [18]. Even if decay may raise an important pulpitis and pain even in third molars, this concept is not often reported in the international literature; therefore, pericoronitis appears to be the first etiology of pain for third molars. Orofacial pain represents the most common and higher pain in head and neck pathology [19]; in this field of pathologies, pain derived from pericoronitis of the third molar appears to be a strong etiology. Third molar pericoronitis has often been confused or referred to different pathologies, including different tooth or temporomandibular disorders [20]. Toothache is a common referred pathology in orofacial pain. Central nervous system hyperexcitability is known to cause expansion of the receptive fields and the spread and referral of pain [21,22].

The implementation of clinically relevant preoperative pain studies in patients with symptomatic third molars has been advocated over the past years: Rudin et al. in their paper on 38 consecutive patients reported with a multiple regression model that the combination of psychological vulnerability and heat pain perception rendered a predictive model that could account for 15 to 30% of the variance in postoperative pain during resting and dynamic conditions [23]. Yuasa and Sugiura in their very interesting paper reported 153 consecutive extractions of third molars in 140 patients and assessed preoperative and postoperative pain, suggesting age and sex as variables for the facial swelling, and pain with tooth extraction difficulty [24]; these concepts are in line with some previous findings [6,7]. Yuasa and Sugiura, as with Rudin et al., underlined and advocated for the implementation of increased preoperative studies of pain related to third molar pericoronitis, to relate this with the nature and magnitude of postoperative pain [23,24].

In order to lead to a better comprehension of pain derived from third molar pericoronitis, the authors of the present article conducted a retrospective observational study with a morphometrical, analytical evaluation of preoperative pain on 86 patients with symptomatic third molars. The purpose of this research was to evaluate pain intensity as a function of symptomatic tooth site (maxillar or mandibular) and to test the possible relationship between pain intensity and the referred area of pain.

## 2. Materials and Methods

### 2.1. Patients

The study had an observational retrospective design. This study enrolled subjects previously visited and scheduled for outpatient third molar surgery at the Oral Surgery School, Dentistry and Dental Prosthodontics, Department of Medical Biotechnologies, University of Siena. All participants signed an informed consent agreement. For all cases, acute pericoronitis was the indication for surgery.

### 2.2. Inclusion and Exclusion Criteria

Inclusion criteria were the presence of partially impacted third molars, confirmed with a preoperative panoramic radiograph, and preoperative pain.

Exclusion criteria were known neurological disease (such as previous trigeminal or facial nerve injuries), impaired communicative or cognitive disease, diagnosed diabetes mellitus, and oral surgical intervention within 30 days before data collection. The presence of a previous extraction was evaluated if in the past.

### 2.3. Parameters Evaluated

**Figure 1:**
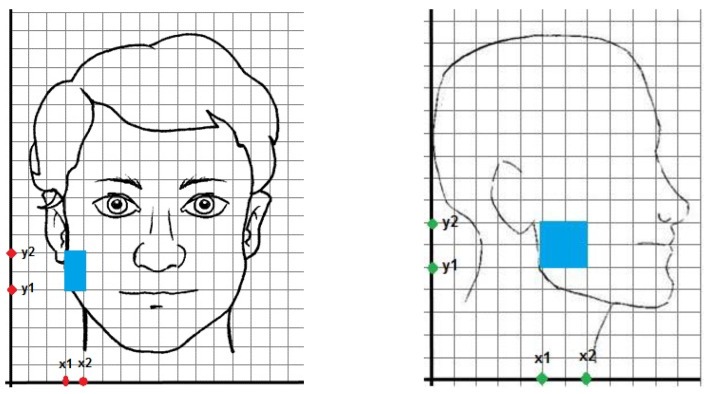
Frontal view of facial graph with a patient pain referral (x_1_ = 3, x_2_ = 4, y_1_ = 5, y_2_ = 7, x-value = 1, y-value = 2) (on the **left**). Lateral view of facial graph with a patient pain referral (x_1_ = 5, x_2_ = 7, y_1_ = 5, y_2_ = 7, x-value = 2, y-value = 2) (on the **right**). With these values, pain area is 6.

Patients were referred to the oral surgery service, Policlinico “Le Scotte”, Siena for third molar pain and requested clinical evaluation. All patients provided a preoperative panoramic radiograph for diagnosis of an impacted third molar and pericoronitis. At the preoperative evaluation, patients were asked to answer a questionnaire about their perception of pain referred to the third molar. The morphometric analytic questionnaire (MAQ) we realized consisted of 5 parts. The first part of the MAQ concerned the possibility that the patient already underwent third molar surgery, as we thought that previous pain sensitization could influence the modulation of pre-operative pain. The second part concerned the third-molar-referred pain as perceived by the patient on a subjective scale from 1 to 10. The third part requested the patient to display over two graphs depicted with a standard face (front section and lateral section) (Figure 1) the perceived pain distribution. Patients were given the full availability of depicting their third molar pericoronitis pain perception as they preferred, with squares or crosses on the image of the affected areas.

All patients received an explanation of their third molar pathology and symptoms after the clinical evaluation, with referral of the maxillary or mandibular third molar. This explanation and clinical evaluation were performed before the filling and compilation of the questionnaire, in order to reduce possible influences on the patient.

Analyses were performed on statistical evaluations regarding patient gender; patient age, both linear and by age range; prior third molar extraction; and site(s) of pericoronitis (maxillar and/or mandible).

**Figure 2:**
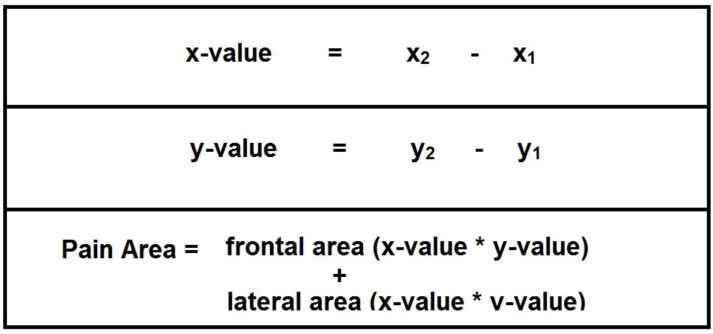
Algebraic formulas for determination of the values.

As it concerns pain parameters, the evaluated items were pain score (1–10) and pain area (defined as sum of frontal and lateral area, expressed as arbitrary units) (Figure 2).

The observational retrospective design did not require the approval of an ethics committee, as per Italian legislation on clinical investigations at the time of the study. Nevertheless, the investigation was carried out following the rules of the Declaration of Helsinki of 1975, revised in 2013, and performed according to the principles of the ICH Good Clinical Practice.

### 2.4. Statistical Methods

All variables were tested for normal distributions (D’Agostino–Pearson test) and data were presented as means with 95% confidence intervals (95% C.I.) for normally distributed variables or median means with 95% C.I. for non-normally distributed data. Differences were evaluated using the independent-sample *t* test (continuous normally distributed data), Mann–Whitney rank sum test (continuous non-normally distributed data), chi-square statistics (categorical variables with minimum number of cases per cell ≥ 5) of Fisher’s exact test (categorical variables with minimum number of cases per cell < 5), one-way analysis of variance (ANOVA), Student–Newman–Keuls post hoc test, or Kruskal–Wallis test. Associations between variables were tested by univariate regression analysis, and two-tailed *p* values of less than 0.05 were considered significant if not otherwise specified. The MedCalc version 11.3.0.0 statistical software package (MedCalc Software, Mariakerke, Belgium) was used.

## 3. Results

All the 86 patients completely filled the questionnaire. A summary of anagraphical data of the patient population is reported in Table 1. 

Between January and October 2019, a total of 86 patients, aged (mean ± SD) 34.54 ± 13.62 years (range 17–78 years), were included in this study: 36 patients were male, aged (mean ± SD) 37.38 ± 14.73 years (range 19–78 years); 50 patients were female, aged (mean ± SD) 32.50 ± 12.51 years (range 17–68 years).

The mean pain score was (mean ± SD) 5.9 ± 2.5, with a mean pain area of 9.9 ± 14.4 (range 2–112). Male patients referred a pain score of 5.9 ± 2.5, with a mean pain area of 12 ± 20.6 (range 2–112). Female patients referred a pain score of 5.9 ± 2.5, with a mean pain area of 8.3 ± 6.6 (range 2–28). 

**Figure 3:**
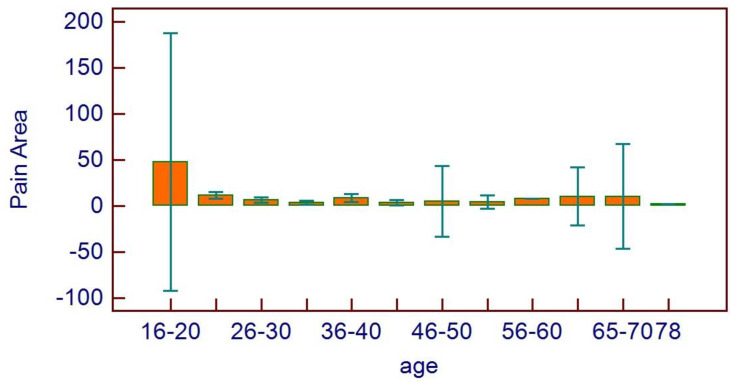
Pain area distribution on the basis of age range.

**Figure 4:**
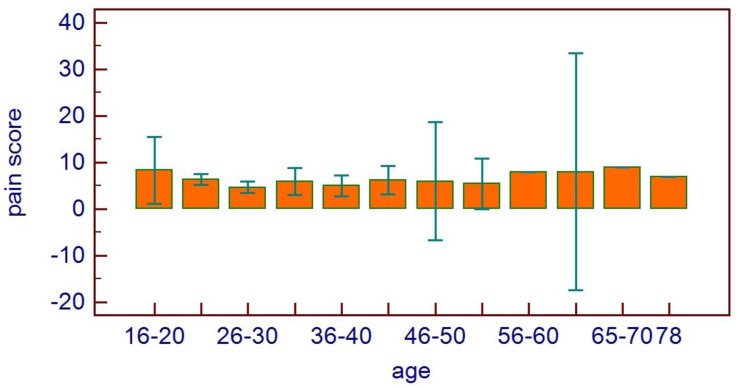
Pain score distribution on the basis of age range.

**Figure 5:**
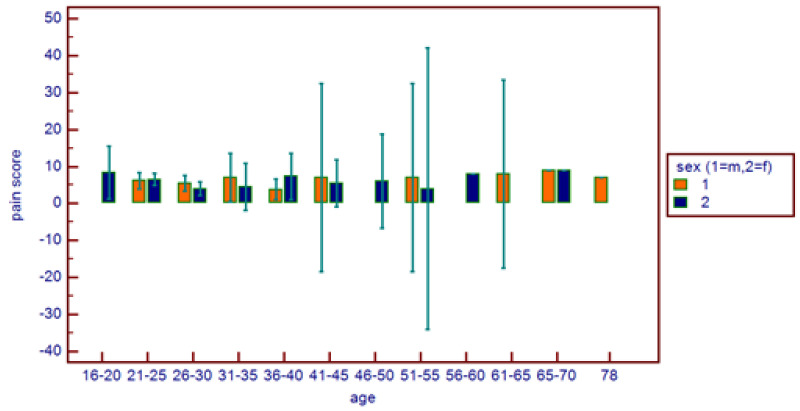
Comparative pain score distribution on the basis of age range and patient gender.

**Figure 6:**
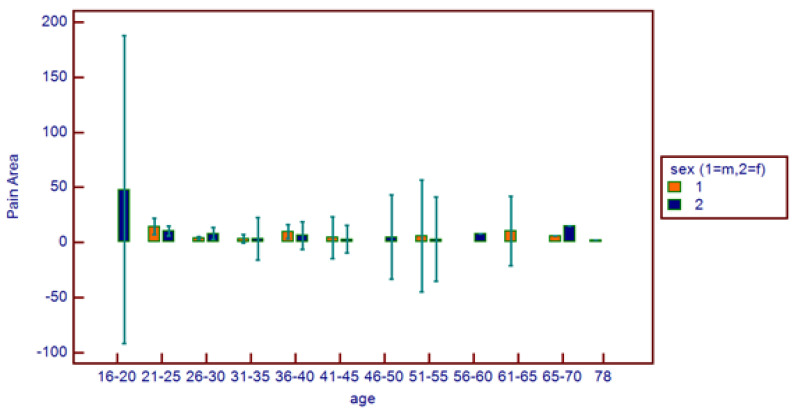
Comparative pain area distribution on the basis of age range and patient gender.

**Figure 7:**
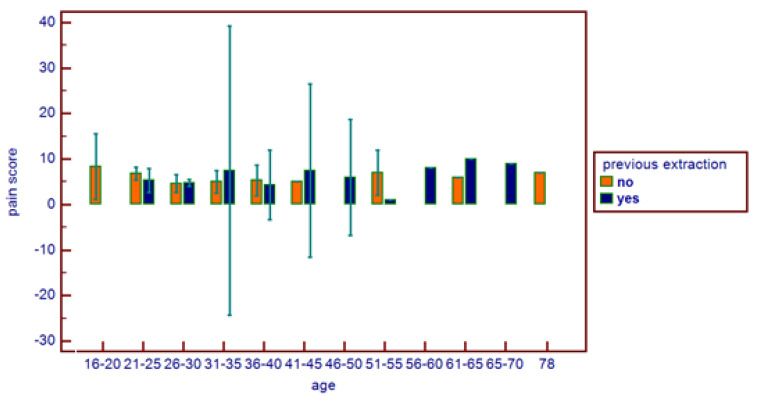
Comparative pain score distribution on the basis of age range and previous extraction.

**Figure 8:**
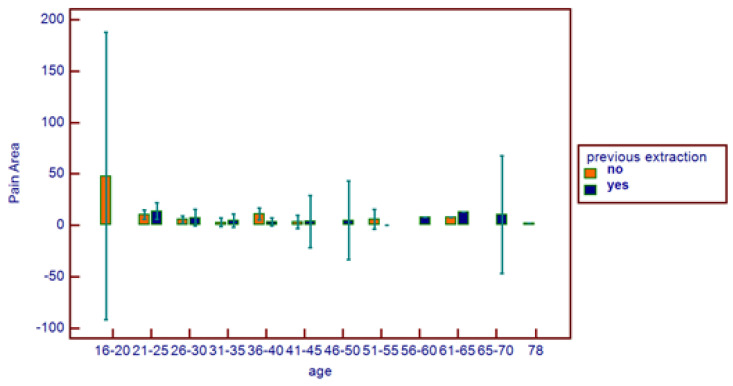
Comparative pain area distribution on the basis of age range and previous extraction.

**Figure 9:**
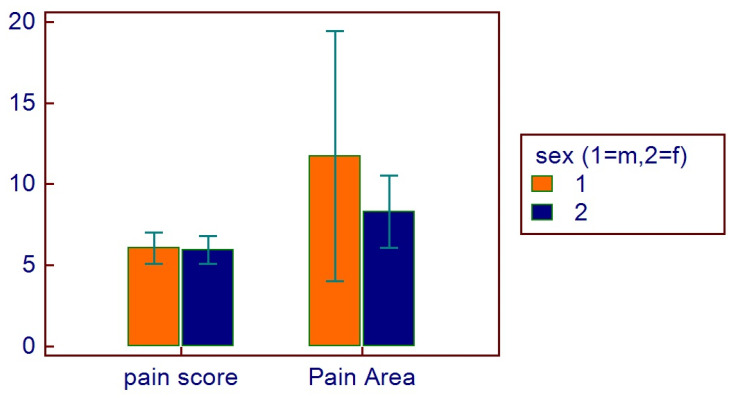
Distribution of pain score and pain area on the basis of sex.

**Figure 10:**
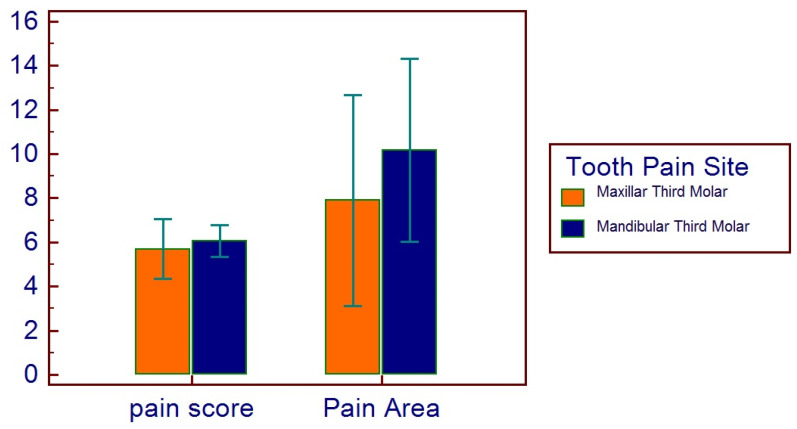
Distribution of pain score and pain area on the basis of tooth pain site, maxillar or mandible.

**Figure 11:**
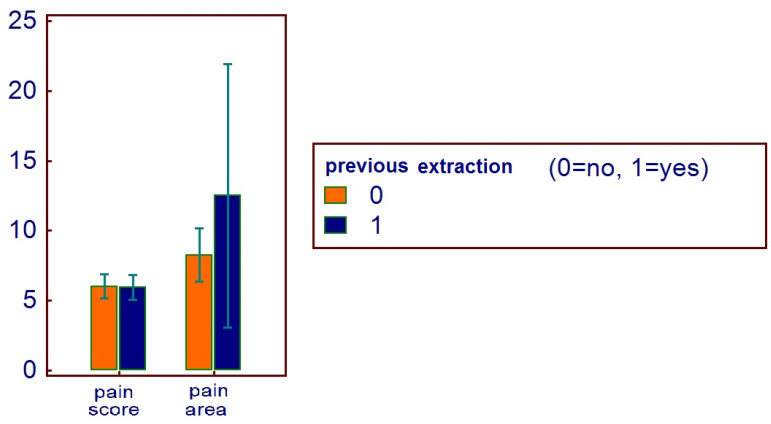
Distribution of pain score and pain area on the basis of previous extraction.

No correlations were found between age and age range with pain area. However, greater values were reported in the 16–20 age range, with reduced values for the greater ranges (Figure 3). Age and pain score parameters resulted in no relations. A flat distribution of pain score was reported (Figure 4). No correlations were found between age and age range with pain score on the basis of patient gender (Figure 5), nor between age and age range with pain area on the basis of patient gender (Figure 6). No correlations were found between age and age range with pain score on the basis of previous extraction (Figure 7), nor between age and age range with pain area on the basis of previous extraction (Figure 8). No statistical differences were observed on pain score in female and male patients. Pain area was higher in male patients than female patients; however, no significant difference was observed (Figure 9). Tooth pain site did not significantly influence pain score or pain area, even if mandibular third molars reported a higher pain than maxillary third molars (Figure 10). The presence of a previous extraction did not significantly influence pain area or pain score, even if pain area was higher in patients with a previous extraction (Figure 11). 

**Figure 12:**
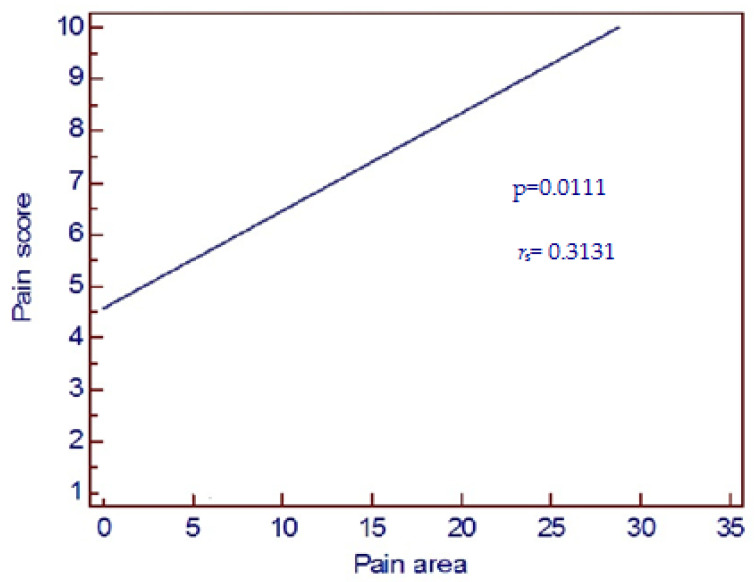
A significant correlation was found between pain score felt by patients and pain area represented on patient’s face (univariate regression analysis *p* = 0.0111, *r*_s_ = 0.3131).

**Figure 13:**
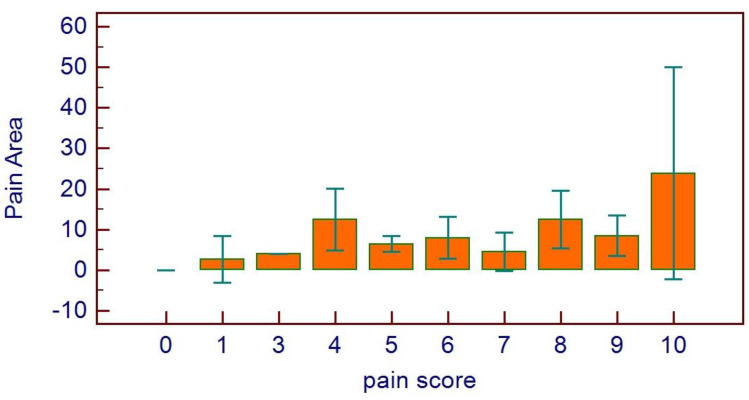
Distribution of pain score on the basis of pain area.

A significant correlation was found between pain score and pain area (*p* = 0.0111, *r*_s_ = 0.3131, Figure 12). Pain score distribution on the basis of pain area scores confirmed the previous data (Figure 13).

## 4. Discussion

On the basis of the present research in third molar pericoronitis, pain intensity indeed resulted in some kind of responsibility in determining the orofacial distribution of pain. The findings of the present article regarding third molar preoperative pain due to pericoronitis confirm earlier data on the relationship between pain severity and referred pain [25]. In addition, the present research also lets us observe that the facial extension of referred pain (pain area) was associated with pain severity (pain score); these results underline and exalt a possible role of the previous research of Falace et al. [21]. On the basis of the international literature at the time of this article, this is the first morphometrical analytical study on wisdom toothache due to pericoronitis, which introduces the association between the facial extension of referred pain and pain severity. 

In this particular setting, referred pain appears to be quite limited in the ipsilateral facial region. While the association of area and pain intensity is in line with Falace et al., the results of the present article of pain location are in apparent contrast with previous reports of toothache by Falace et al. and Wolff et al., who reported a wide dispersion of pain [21,25]; Wilson et al. reported that high levels of somatization with a high pain intensity significantly predicted a wide dispersion of referred pain [26]. However, all the findings from Falace et al., Wolff et al., and the present article are based on reports of pain ranging from days to one month, while Wilson et al. reported chronic orofacial pain conditions lasting years [21,24,25]; for this reason, Wilson et al.’s pain somatization differs from the previous results, and cannot be the reason for the difference between our data and those of Falace et al. and Wolff et al. However, somatization is surely a paradigm that should be considered in differential diagnosis. On the other hand, individual hyperexcitability may not explain this discrepancy, as our patients did not receive oral intervention 30 days before collecting MAQ data; hyperexcitability has been recognized to last for up to 30 days after oral surgery [27].

The referral patterns of pain areas reported in the present study could be assumed as a pain model resulting from pericoronitis of the maxillar and mandibular third molars. The present results confirm previous data on the prevalence of younger patients (20–30 years old) seeking third molar extraction due to orofacial pain [28]. As compared with previous mapping reports of toothache referral patterns, the data of the present article indicate that pain arising from third molar pericoronitis appears to be quite limited to the ipsilateral facial area, with an almost overlapping extension for the maxillar and mandibular third molar [21].

In this study, no correlations were found between age, gender, previous extraction, and tooth site, while the results suggested a strong influence of pain score on orofacial pain distribution (pain area). This influence of high pain that leads to a diffuse orofacial distribution of pain could let the authors speculate on possible influences of postoperative recovery after third molar surgery [2,3,4,5,6,7]. So, while postoperative pain may be influenced by patients’ individual characteristics, preoperative pain and the orofacial distribution of preoperative pericoronitis pain were independent from these characteristics, with the limits of this study. Mobilio et al. in a 2011 research paper reported good results with a preoperative pain evaluation test to identify patients at risk of developing greater pain after third molar surgery. Mobilio reported that patients at risk of developing greater postoperative pain are characterized by a higher level of reported preoperative pain or unpleasantness after exposure to a nociceptive stimulus. Further, Mobilio suggested that his test may be tailored to specific patient needs for postoperative treatment [5]. On the other hand, Hosgor et al. in their interesting paper regarding third molar preoperative pain suggested that preoperative pressure pain threshold, pressure pain tolerance, and anxiety level had no significant effects on postoperative pain and analgesic requirements in impacted lower third molar surgery [29]; this concept contradicts the article of Mobilio et al. and represents a large gap between two interpretations of third molar pain and pericoronitis. It is important to consider that pain, especially pathological pain, has characteristics that can be influenced by psychopathological aspects of the patient. Therefore, it is important to deepen the study of the pathological pain of pericoronitis of the third molar in order to better measure and evaluate the relationships between preoperative and postoperative pain [30]. In this study, the pain area was greater in the 16–20 and 65–70 years old range, even if with no statistical significance. Further, with regard to the variable of previous extraction of the patients, higher values were shown irregularly. Male patients experienced increased pain area compared to the female patients, even if not statistically significant. A previous study reported instead a significant gender difference in oral-health-related quality of life after mandibular third molar surgery. The perceived pain in this study was limited to the preoperative one, but as reported by many authors, the development of an accurate knowledge of preoperative pain may lead to an increased understanding of postoperative pain and oral disability after mandibular third molar surgery [6,23,24]. With regard to gender influence, Silva et al. reported opposite results to previous studies, reporting that pain, edema, and trismus after impacted third molar extraction were not influenced by gender; this information is still different from other studies and reports only to postoperative pain [31].

The main limits of this paper are the exclusive study of preoperative pain, without examination of postoperative recovery. Further, even if 86 patients represent an interesting number for statistics, the age and patient gender were not completely equally matched; this limit especially was felt in the age range evaluations of pain area pain score, where in some ranges, the distribution of patient gender was not homogeneous. Further, the greater presence of the mandibular third molar over the maxillary third molar impacted the study in some ways. Diabetes mellitus was reported among the exclusion criteria from this retrospective study. Diabetes mellitus is an endocrine disease with documented evidence of interference in nerve sensitivity and transmission that takes the name of diabetic neuropathy [32]. As far as the oral cavity is concerned, a worsening of the quality of life in healing after tooth extraction is documented in patients with diabetes, and some authors suggest a correlation between third molar pathology and diabetes mellitus in some patients [33,34,35]. Therefore, to avoid bias related to erroneous assessments of preoperative pain in diabetic patients, we excluded these patients from this study and previous studies [36].

On the basis of this research, the authors suggest different characteristics of preoperative pain to investigate. Future prospectives of third molar pericoronitis pain research should begin with the examination of different possible pathologies of the follicular sac surrounding unerupted third molars. We advocate the use of the histological study of the follicular sac and pericoronitis symptoms with evaluation of the state of pericoronitis. The study of these preoperative conditions could be correlated with postoperative conditions, such as postoperative non-pain complications, swelling, and infection. For this purpose, a recent study underlined a different bacterial retention on sutures after third molar surgery, and we advocate studies of pericoronitis and postoperative bacterial retention [36].

Further, the study of preoperative pain versus no pain in third molar impaction should be investigated in order to relate with postoperative pain; this information could be useful for the clinician in order to counsel properly patients with regard to oral disability after tooth extraction [37].

## 5. Conclusions

The present research study investigated and reported the intensity, distribution, and correlations between variables of preoperative-third-molar-related pain. The results of the present article allow the authors to link the third molar preoperative pain and the third molar orofacial distribution of preoperative pain by a direct correlation.

## Figures and Tables

**Table 1:** Anagraphical data of the patients (SD = standard deviation).

Patients	86
mean age ± SD	34.54 ± 13.62
M/F	36/50
previous extraction Y/N	24/62
maxillar third molar	18
mandibular third molar	68
pain score ± SD	5.9 ± 2.5
pain area ± SD	9.9 ± 14.4

## Data Availability

No new data created.
